# Cash transfers targeting adolescent wellbeing: a scoping review of the literature in low- and middle-income countries

**DOI:** 10.1080/16549716.2026.2691385

**Published:** 2026-06-24

**Authors:** Simone H. Crouch, Andrea Kolkenbeck-Ruh, Kathleen Kahn, Lisa K. Micklesfield, Shane A. Norris

**Affiliations:** a SAMRC/Ageing African Adult Research Unit, Department of Paediatrics, Faculty of Health Sciences, University of the Witwatersrand, Johannesburg, South Africa; b Cardiovascular Pathophysiology and Genomics Research Unit (CPGRU), Department of Physiology, School of Biomedical Sciences, Faculty of Health Sciences, University of the Witwatersrand, Johannesburg, South Africa; c SAMRC/Wits Rural Public Health and Health Transitions Research Unit (Agincourt), School of Public Health, Faculty of Health Sciences, University of the Witwatersrand, Johannesburg, South Africa; d School of Human Development and Health, University of Southampton, Southampton, UK

**Keywords:** Social protection, nutrition, mental and sexual health, school attendance, behaviour interventions

## Abstract

Creating healthy food environments is central to improving adolescent nutrition; a life-stage characterised by rapid growth, body composition changes and heightened nutrient requirements. In low- and middle-income countries (LMICs), cash transfer (CT) programmes are increasingly implemented as poverty-alleviation strategies to influence diet quality and related health behaviours by relaxing household budget constraints. While evidence demonstrates CT effects on nutrition and health in infancy and early childhood, few studies have evaluated their impact in adolescents in LMICs. We conducted a scoping review of randomised controlled trials and quasi-experimental studies (2012–2025) evaluating CT interventions reporting adolescent (10–19 years) outcomes in LMICs. Comprehensive searches of PubMed and Google Scholar yielded 164 publications, of which 19 were included. Studies were eligible regardless of whether the CT programme targeted adolescents, provided adolescent-related outcomes were reported. Outcomes were categorised by: mental health; school attendance/enrolment; HIV prevalence; sexual activity/behaviour; partner violence; economic and food security; tuberculosis treatment; nutritional status; and healthcare utilisation. Almost all trials were conducted in sub-Saharan Africa (SSA), eight studies examined the effect of conditional cash transfer (CCT), five studies unconditional cash transfers (UCT), five studies both CCT and UCT, while one study reported ‘cash transfers’ with no further information on the type of CT. Most studies reported beneficial effects in domains closely linked to the social determinants of adolescent health (schooling, economic security, sexual and reproductive health, and psychosocial wellbeing). In conclusion, CTs can contribute to improved adolescent wellbeing in LMICs, however direct evidence on adolescent nutrition outcomes is sparse.

## Paper Context

- **Main findings:** Findings suggest cash transfer programmes improve several aspects of adolescent wellbeing, particularly schooling participation, economic security, and sexual and reproductive health risk, but provide little direct evidence of improved nutrition outcomes.
- **Added knowledge:** This review highlights that a critical evidence gap remains regarding the effects of cash transfer programmes on adolescent nutrition outcomes, as nutrition indicators were rarely assessed in trials.
- **Global health impact for policy and action:** Policies and programmes should prioritise the design and evaluation of nutrition-sensitive cash transfer interventions for adolescents, using consistent nutrition indicators and longer-term follow-up to guide scalable strategies to address malnutrition in low- and middle-income countries.

## Background

Chronic malnutrition and poor health in both childhood [1] and adolescence [2] have severe long-term consequences, including an increased risk of cardiovascular disease and reduced social and/or economic productivity in adulthood [3]. Despite this, adolescent health, particularly nutrition, has received relatively limited attention in government policies and programmes. Highlighting this gap, the recent Lancet Adolescent Nutrition series [2,4,5] emphasised adolescence as a critical window for growth and development, highlighting the importance of nutrition for physiological health, developmental outcomes, and the health of the next generation [2]. Adolescent malnutrition remains a significant barrier to health and human capital development worldwide. Evidence from the Global School-Based Student Health and Health Behaviour in School-Aged Children surveys indicated that 10.2% of adolescents aged 12–15 years in low- and middle-income countries (LMICs) were stunted, 5.5% were underweight, and 21.4% were overweight or obese [6]. Nutritional shifts are rapidly occurring in LMICs, as a result of urbanisation, economic transition and shifts in the food industry, driving increasing levels of adolescent obesity, despite the continued persistence of undernutrition [3,7]. This double burden contributes to poor adolescent health outcomes that often track into adulthood.

Chronic malnutrition is multidimensional, shaped by various underlying factors, including poverty and social vulnerability [8]. Programmes and policies around the world have attempted to address this long-standing issue, with one widely implemented strategy being the use of conditional cash transfer (CCT) programmes. First introduced in Latin America as part of poverty reduction programmes and later extended to sub-Saharan Africa (SSA) [9], CCTs provide financial support to poor and vulnerable households, conditional upon compliance with requirements including the utilisation of maternal and child health services, and school enrolment and/or attendance [10]. Although initially designed to reduce poverty and improve education and health service utilisation, CCTs may also influence adolescent health and nutrition through multiple pathways.

**Figure 1.**
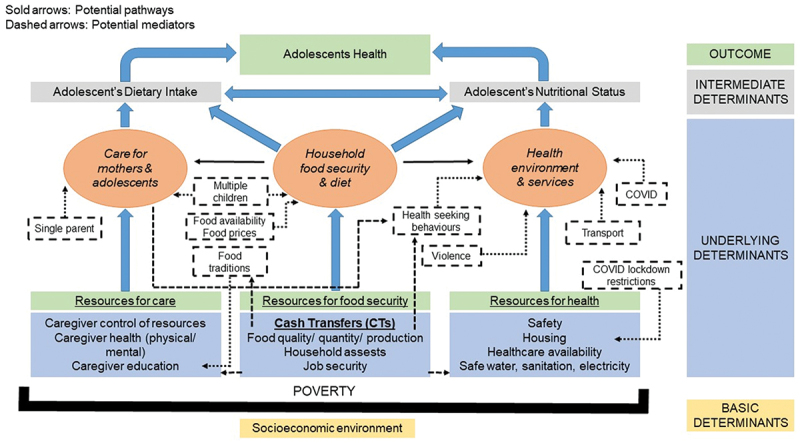
Adaptation of de Groot et al [11] conceptual framework of the determinants of adolescent health.

Figure 1, adapted from de Groot et al. [11], illustrates how the addition of financial resources through cash transfers may influence the underlying determinants of child and adolescent health via three interconnected pathways: food security, health environment, and care. These underlying determinants in this framework interact to shape adolescents’ overall health status, with particular emphasis on household food security and diet, given their central role in nutritional status.

In this model, *household food security* refers to the availability of sufficient resources to ensure adequate food consumption for all household members. These resources may derive from cash transfers (including social grants or CCT programmes), earned income through employment, or household food production. Household assets, such as refrigeration for food storage, alongside income or production capacity, influence both food quantity and quality. *Care* encompasses caregiver behaviours that affect adolescent health and development, including psychosocial care, feeding routines, food preparation, health-seeking behaviour, and healthcare. Care for mothers and adolescents is determined by caregiver control over resources, caregiver mental and physical status (levels of stress, caregiver nutritional status, and overall health), knowledge/education, preferences and/or beliefs (food traditions). The third underlying determinant, *health environment*, reflects the mothers’ and adolescents’ access to and availability of safe water and sanitation facilities, healthcare, and shelter.

Within this framework, cash transfers may influence adolescent health through three principal pathways. First, cash transfers directly increase household disposable income and, consequently, the resources available for improved household food security. If households use the cash to purchase a higher quantity or quality of food, or invest in food production or productive assets, household food security and household diet diversity may improve. Second, cash transfers affect the household level resources for health, where increased resources may allow for improvements to sanitation facilities in the home or housing conditions, reducing the risk of diseases and infections. Third, cash transfers can increase access to healthcare by supporting out-of-pocket expenses such as transportation to health facilities, consultation fees, medical supplies, preventive medicines, or essential vaccinations (e.g. COVID-19). Collectively, these mechanisms may contribute to improved adolescent health outcomes.

Evidence to date suggests that CCTs improve the utilisation of maternal and child health services, however findings regarding the impact on child health and nutritional status remain mixed [11–14]. Cash transfer programmes can also serve as delivery platforms for other nutrition interventions [9], such as behaviour change communication, which has demonstrated improvements in knowledge and practices around child feeding, and to a lesser extent, anthropometric outcomes [15–19]. However, much of the research evaluating CCTs and nutrition has focused on infancy and early childhood (<5 years) nutrition [1,20–33], and less on adolescent health and nutrition. Thus, this paper had two aims: first, to scope and synthesise the evidence related to the impact of CCTs on adolescent outcomes, specifically in low- and middle-income countries and second, to examine the extent to which these interventions addressed nutrition outcomes.

## Methods

**Figure 2.**
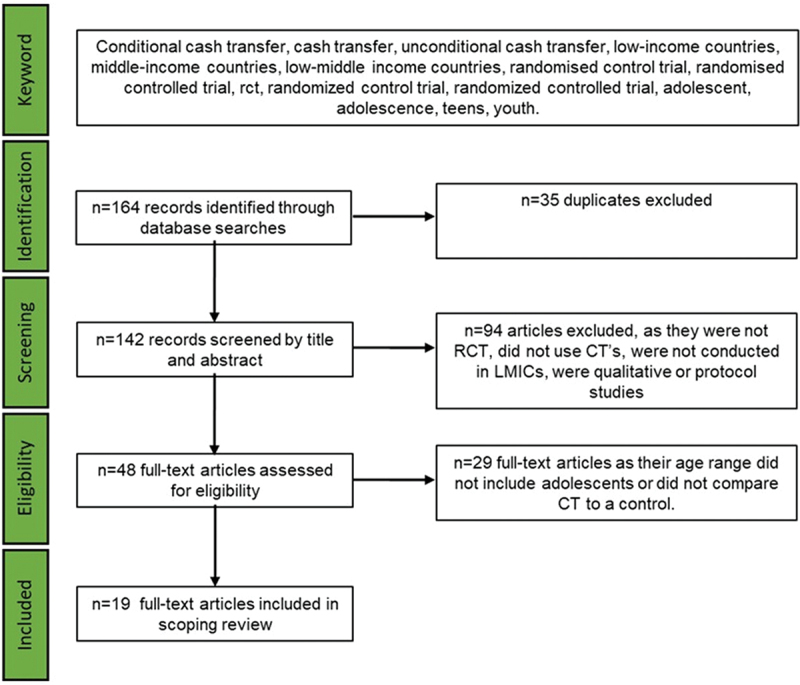
A flow diagram depicting the study selection criteria.

We conducted a scoping review of the literature examining the effect of cash transfers on adolescent outcomes according to the guidelines of PRISMA-ScR for systematic reviews and meta-analyses extension for scoping reviews [34] as shown in Figure 2. We defined adolescence as 10–19 years of age [35].

### Data searches and search strategy

We conducted a broad search of the literature using PubMed and Google Scholar with selected terms. Search terms were identified to assess randomised control trials examining the impact of cash transfers (conditional and unconditional) targeting adolescent health in low- and middle-income countries. An overview of the search strategy was as follows: *(Conditional cash transfer OR cash transfer OR unconditional cash transfer) AND (low-income countries OR middle-income countries or low-middle-income countries) AND (randomised control trial or randomised controlled trial or rct or randomized control trial or randomized controlled trial) AND (adolescent OR adolescence OR teens OR youth)*. A review of titles and abstracts was conducted to ascertain the relevance of the selected articles for the scoping review. The search was conducted in January 2026, including all studies conducted from 2012 onwards. Within the articles presented in this scoping review, we highlight the findings from randomised controlled trials conducted in LMICs, using cash transfers (conditional and unconditional) to improve adolescent health. All retrieved studies were exported to a web-based screening software tool, Rayyan (http://rayyan.qcri.org) [36], for title and abstract screening, duplicate detection and full text screening. Search, abstract and full text screening were conducted by SHC and AKR; screening was conducted in duplicate. Data extraction was conducted by AKR and reviewed by SHC.

### Inclusion and exclusion criteria

We included individual and cluster randomised controlled trials and quasi-experimental trials conducted from 2012 onwards. Only studies published in English that examined cash transfer interventions focused on adolescent outcomes (ages 10–19 years), or included adolescents within this age range, in low- and middle-income countries were included in the scoping review. Studies were eligible regardless of whether the cash transfer programme specifically targeted adolescents, provided adolescent-related outcomes were reported. No restrictions were applied based on cash transfer programme characteristics such as transfer size, duration, conditionality, or delivery mechanism. Both two-arm and multi-arm randomised trials were eligible for inclusion. We excluded studies that only examined specific subsets of the population (e.g. children or adolescents with diabetes or in hospital settings), as well as systematic reviews, qualitative studies, and study protocols.

## Results

The search yielded 164 studies, 35 of which were deleted as duplicates. One hundred and forty-two studies were screened by abstract and title, of which 94 were excluded. Forty-eight articles were then full-text screened, and 29 were excluded due to participant age range not including adolescents and studies not comparing a CT to a control group. The remaining 19 studies were included in this review (Figure 2). The included studies were published over thirteen years, between 2012 and 2025. The majority of the studies were from Africa (*n* = 18: 5 in Kenya [37–41]; 4 in South Africa [42–45]; 3 in Malawi [46–48]; 2 Tanzania [49,50]; 2 in Zambia [51,52]; 1 in Zimbabwe [53]; 1 in Liberia [54]), while one study was conducted in Peru, South America [55].

### Key findings from the studies

Table 1 summarises the characteristics, type of cash transfer (CT) interventions, and outcomes of the 19 studies included in the scoping review. Eight studies examined the effect of conditional cash transfers (CCT), while five studies looked at the effect of unconditional cash transfers (UCT) on adolescent health. Five studies looked at both CCT and UCT on adolescent health, while one study reported the use of ‘cash transfers’ on adolescent health, with no further information on the type of CT. Of the included studies, 89.5% (*n* = 17) found that the cash transfer intervention had a positive effect on aspects of adolescent wellbeing; however, the outcomes measured varied, while two studies reported no effect of cash transfers on the outcome. Studies were categorised according to the following adolescent health outcomes: mental health and depression; school attendance/enrolment; HIV prevalence; sexual activity/behaviour; partner violence; economic and food security; tuberculosis treatment; nutritional status; and healthcare utilisation.

**Table 1.** Characteristics of included studies (*n* = 19).

*Author*	*Country*	*n*	*Age range (years)*	*Intervention*	*Duration of Intervention*	*Size of & Frequency of CT*	*Primary outcome*	*Findings*	
								*Primary outcome effect* *To what extent*	
							*Secondary outcome*	*Secondary outcome effect* *To what extent*	
Austrian et al. [41]	Kenya	2147	11–14	Violence prevention (V-only) V-only and education (VE) (CCT) VE and health (VEH) (CCT) all four interventions (VEHW) (CCT)	2 years	Education (VE) ~10% of monthly household expenditure for primary school beneficiaries Two cash transfers to household head per term (~$15 per transfer) 1st transfer upon enrollment 2nd transfer upon verified continued attendance School supply kit for girl upon enrollment each term (value ~$6) School fees partially covered upon enrollment each term (up to ~$7 for primary and ~$60 for secondary) Incentive to school upon enrollment each term (~$5 for each AGI-K girl enrolled) Wealth Creation (VEHW) Provided home banks/piggybanks Annual savings incentive: ~$3	Been married; been pregnant; given birth	Yes	When compared to V-only, in participant out of school at baseline in VE and VEHW but not VEH there was a positive impact on ever married and has ever been pregnant (±0.4SD)
							None		Not applicable
Kangwana et al. [40]	Kenya	2075	11–14	Violence prevention (V-only) V-only and education (VE) (CCT) VE and health (VEH) (CCT) all four interventions (VEHW). (CCT)			Had sex; Been pregnant; Given birth; Herpes simplex virus-2.	Yes	Ever having given birth was reduced by 2.3% points (pp) in the VE and VEHW study arms, significant at 10%. For the older subsample, there were significant reductions in the percent ever having had sex (8.2 pp), HSV-2 prevalence (7.5 pp) and HSV-2 incidence (5.6 pp) in the VE arm.
							Education, health knowledge, and wealth creation	Yes	When compared to V-only, in a pooled sample of VE, VEH and VEHW there was a positive effect on education, health, and wealth summary outcomes of between 0.1 and 0.2 SD.
Ayuku et al. [37]	Kenya	1481	0–18	UCT	5 years	A cash payment of KSH. 1500/= (approximately $17 USD) per month paid every two months through the Kenya Post Office or Equity Bank	School enrolment, nutritional status	Yes	CT groups were less likely to have missed any days of school in the preceding month 1–18 years were less likely to have height stunting for their age
							None		Not applicable
Handa et al. [38]	Kenya	1811	15–25	UCT	2 years	A flat transfer of approximately USD20 (at an exchange rate of US$1:KES75 in 2007) per month (paid bi-monthly) given directly to the caregiver.	Sexual activity	Yes	Those in treatment households had significantly lower odds of having initiated vaginal intercourse since baseline than young people in control households.
							Condom use, number of partners and transactional sex.	No	There were no statistically significant effects on condom use, number of partners and transactional sex.
Handa et al. [39]	Kenya	1547	12–24	UCT			Pregnancy and early marriage among females	Yes	The programme reduced the likelihood of pregnancy; there was no significant impact on the likelihood of early marriage.
							None		Not applicable
Baird et al. [46]	Malawi	1706	13–22	CCT, conditional of school attendance UCT	2 years	On a monthly basis for a total of ten transfers per year. In the conditional group, each payment was received if the girl attended school for 80% of the days that school was in session during the previous month. In the unconditional group payment was received if the girl attended the cash transfer points. Cash transfers were split between guardian and girl. Household amount varied randomly (by use of computer-generated random numbers) by enumeration area, with monthly values of US$4, $6, $8, or $10. Girl amount varied randomly between individuals, with monthly values of $1, $2, $3, $4, or $5, decided by drawing numbers from an envelope.	Prevalence of HIV and herpes simplex virus 2	Yes	•HIV: Intervention group 1.2% vs 3.0% in the control group HSV-2: Intervention group 0.7% vs 3.0% in the control group
							Self-reported sexual activity, school enrolment	Yes	•Self-reported sexual activity was significantly lower in the intervention group. Individuals in the intervention group were enrolled in school.
Beauclair et al. [47]	Malawi	2907	13–22	UCT; CCT, conditional at least 80.0% of school days			Effect of relationship age difference on condom use and sex frequency. The effect of relationship age difference on relationship duration	Yes	Girls receiving CT had smaller age differences in relationships compared to controls, was not statistically significant The older the participant was, the smaller her age differences Across treatment groups, larger age differences in relationships were associated with lower levels of condom use, more frequent sex, and longer relationship durations.
							None		Not applicable
Ozler et al. [54]	Liberia	1176	13–14	Girl Empower [GE] (life skills) GE+ (above + CT tied to girls’ participation in weekly sessions)	2 years	GE All participating girls received cash to help start their own savings account, along with a savings book and a cash box. Each girl received $2 per month for a total of $16 during the eight-month implementation period. GE+ Caregivers of programme participants received of a payment of $1.25 for each of the 32 regular sessions that the adolescent girl attended, for a maximum of $40. Once the previous month’s attendance was known and confirmed for each participant	Sexual violence, schooling, sexual/ reproductive health; psychosocial wellbeing, gender attitudes, life skills and protective factors	Yes	The effects of both GE and GE+, compared to control, on sexual violence, schooling, psychosocial wellbeing, and protective factors were small and not statistically significant. Found positive significant standardised effects on Gender Attitudes, life skills, sexual/ reproductive health
							None		Not applicable
Wingfield et al. [55]	Peru	282	5–19	SOC SOC + CCT on condition with regular meetings	6 months (extendable)	Cash transfer incentives were stratified into ‘double’ and ‘simple’ incentives. Double incentives were made for meeting the condition ‘optimally’ (i.e. monthly adherence missing less than two daily tablets) at 230 USD. Simple incentives were made for meeting a condition ‘acceptably’ (i.e. monthly adherence in which two or more tablets had been missed but the patient had not abandoned treatment) at 115 USD. In situations in which TB treatment routinely extended beyond 6 months, such as HIV-TB co-infection (9 months) or multi-drug resistant (MDR) TB (18 to 24 months), cash transfers continued throughout the duration of treatment.	Initiation of TB preventive therapy	Yes	43.0% in the intervention arm initiated tuberculosis preventive therapy versus 25.0% in the control arm
							Successful TB treatment	Yes	Treatment was successful in 64.0% (87/135) of patients in the intervention arm versus 53.0% (78/147) in the control arm
Cluver et al. [42]	South Africa	3401	10–18	CT	1 year	Receipt of one of the following: he child support grant (ZAR250 per month in 2010, ZAR280 per month in 2012; roughly equivalent to US$35), available to all primary caregivers of children who earn less than a means-tested benchmark or foster child grant (ZAR710 per month in 2010, ZAR770 per month in 2012; US$96), available to primary caregivers of a child legally in their care, as a result of being orphaned, abandoned, at risk, abused, or neglected	Prevalence of transactional sex, age-disparate sex, unprotected sex, multiple partners, and sex while drunk or after taking drugs	Yes	In girls, CT was associated with reduced incidence of transactional sex and age-disparate sex with similar associations for prevalence. In girls, no significant effects were shown for other risk behaviours. In boys, no consistent effects were shown for any of the behaviours.
							None		Not applicable
Kilburn et al. [43]	South Africa	2448	13–20	CCT, conditional on monthly school attendance	3 year	Participants and their parents or guardians received monthly cash transfers of 100 and 200 Rand (R) respectively (or roughly US$ 10 and US$ 20 using 2012 the conversion rates). Cash transfers were conditional on the young woman attending at least 80% of school days during the month. As long as the young woman was eligible to be in school and met the attendance criteria, she could receive the transfer for up to 3 years.	Intimate partner violence (IPV)	Yes	•CT reduces sexual debut and sexual partners in the previous 12 months. •IPV was reduced, potentially due to the effect of the reduction in sexual debut and sexual partners in the previous 12 months.
							None		Not applicable
Kilburn et al. [44]	South Africa	2448	13–20	CCT, conditional on monthly school attendance			Depression	Yes	Interaction terms show the poorest young women had higher sexual relationship power scores, greater hope and lower depressive scores.
							Economic and food security		CCT significantly increased both total and food per capita household consumption but showed no significant differential effect by baseline poverty status.
Pettifor et al. [45]	South Africa	2448	13–20	CCT, conditional on school attendance			HIV incidence	No	HIV incidence did not differ between those who received a CT and those who did not
							None		Not applicable
Prencipe et al. [49]	Tanzania	880	14–28	CCT CCT Plus		Fixed benefit: 10,000 TZS (5 USD) 4,000 TZS (1.80 USD) additional with any children under 18 years Conditional benefit: 4,000 TZS (1.80 USD) contingent on health compliance for children under 5 years (flat rate) 2,000 TZS (0.90 USD) contingent on primary school enrollment (up to 4 children) 4,000 TZS (1.80 USD) contingent on lower secondary school enrollment (up to 3 children) 6,000 TZS (2.70 USD) contingent on upper secondary school enrollment (up to 2 children)	Depression / depressive symptoms	Yes	In males, a significant reduction in depressive symptoms In females, depressive symptoms increased. Females who were 18+ years and with children drove this negative effect
							None		Not applicable
Kuringe et al. [50]	Tanzania	2720	15–23	Combination HIV prevention (CHP) CHP+ UCT	18 months	UCT in quarterly instalments of 70,000 Tanzania shillings (~US $31) for 18 months through mobile money on a project-provided cellular phone.	Herpes simplex virus-2	Yes	No significant difference in HSV-2 incidence between the study arms. Location significantly modified the effect, in rural areas the incident HSV-2 infection in the CT group was significantly lower.
							HIV prevalence, intergenerational sex, and transactional or compensated sex, use of biomedical services.	Yes	No significant difference in HIV prevalence. The CT intervention reduced the proportion of transactional sex, increased savings, and increased utilisation of community-based biomedical services. No significant difference in the proportion reporting compensated and intergenerational sex, sex work, and more than one sexual partner in the last 12 months. Urbanisation modified the effect of CT intervention on savings, utilisation of biomedical services, and sex work. In urban areas, CT was associated with a significant increase in reporting of sex work.
Schaefer et al. [53]	Zimbabwe	2909	15–59	CCT, conditional on school attendance UCT	1 year	In the UCT programme, households collected $18 plus $4 for each child in the household (up to a maximum of $30) from pay points every 2 months. In the CCT programme, households received the same amount if they met several conditions: applied for a birth certificate for children not yet registered within 3 months; kept children under 5 years up-to-date with vaccinations and attended growth-monitoring sessions twice a year; children aged 6–17 years attended school at least 90% of the time in a month; and one person from the household attended at least two-thirds of a local parenting skills class.	Sexual behaviour	Yes	CTs reduced having any recent sex among young males and females, with similar but less uncertain estimates when compared against the synthetic comparison group. There were no effects among older individuals. Young (but not older) males receiving CTs reported increased multiple partnerships
							Mental distress, school enrolment, alcohol/drug/ cigarette consumption	Yes	No impact of alcohol, cigarette, or drug consumption was found. CTs increased school enrolment in males and females and condom use among younger and older women receiving CTs
Hegdahl et al. [51]	Zambia	4922	Grade 7–18years	Economic Support (ES): UCT adolescent + UCT parent + conditional school fees (CCT) ES + community dialogue	2 years	The economic support arm consisted of a monthly CT of ZMW 30/month (USD 3 at the time of intervention) to the participating girls, an annual CT of ZMW 350 (USD 35) to their parents/guardians, as well as payment of school fees for girls continuing to grades 8 and 9. Payment of school fees was done directly to the school bank accounts. Girls who dropped out of school could continue to receive the economic support until age 18.	Sexual activity and contraceptive use and knowledge	Yes	Sexual activity in the previous four weeks was lower in the ES arm compared to the control, and in the combined arm compared to the ES arm. Unprotected sexual activity was lower in the ES arm compared to the control and substantially lower in the combined arm compared to the two other arms. A higher proportion of girls reported current contraceptive use in the combined than in the ES arm and control. The proportion of girls reporting recent contraceptive use was similar across the three groups. Knowledge of modern contraceptive methods was similar in the control and ES arms at the end of the intervention period. Compared to ES alone, a higher proportion had good knowledge among those in the combined intervention.
							None		Not applicable
Mori et al. [52]	Zambia	4922	Grade 7–18 years	Economic Support (ES) UCT adolescent + UCT parent + conditional school fees (CCT) ES + community dialogue			Healthcare utilisation	No	Economic support alone and in combination with community dialogue did not have a substantial impact on healthcare utilisation.
							Catastrophic health experiences	No	Economic support alone and in combination with community dialogue did not have a substantial impact on catastrophic health experiences.

#### Mental health and depression

A study conducted in Malawi (*n* = 2782, 15–22 years) reported a 10–15% improvement in mental health outcomes in those who received a UCT compared to the control group that did not receive a UCT. The UCT programme reduced depression by 15% in girls in the intervention group compared to the control group [48]. Similar results were found in a South African study (*n* = 2448, 13–20 years), where CCT had significant beneficial effects on psychosocial well-being and depression [44]. This was also observed in Tanzania (*n* = 880, 14–28 years), where there was a 1.5% (95%CI: 2.56 to −0.04) reduction in depressive symptoms in males who received a CCT compared to their control counterparts [49]. However, the authors did find a 1.1% (95% CI 0.11;2.09) increase in depressive symptoms in females who were 18 years and older [49].

#### School attendance or enrolment

A study by Ayuku et al. [37] reported that Kenyan adolescents < 19 years (*n* = 1481, 0–18 years) who received a UCT were less likely to have missed any days of school in the preceding month compared to those not receiving the UCT (aOR: 0.32, 95%CI 0.16;0.63). Kangwana et al. [40], (*n* = 2075, 11–14 years) compared a programme of violence prevention only (V-only); V prevention, education and CCT (VE); VE and health (VEH); or all four interventions (VEHW) in Kenyan adolescents. They found that when compared to V-only, the pooled sample of the CCT groups showed a positive effect on z-scores for education (0.13, 95%CI 0.03;0.22), health (0.14, 95%CI 0.04;0.24), and wealth (0.23, 95%CI 0.12;0.33) [40]. Similarly, studies conducted in Malawi (*n* = 1706, 13–22 years) and Zimbabwe (*n* = 2909, 15–59 years) found that those in the intervention group, receiving a cash transfer, were more likely to be enrolled in the school year compared to those who did not receive CTs [46,53].

#### HIV and HSV-2

A study in Malawi (*n* = 1706, 13–22 years) found HIV prevalence was lower in adolescents receiving either a UCT or CCT (on condition of school attendance) compared to those not receiving any cash transfer (1.2% vs. 3.0%) [46]. In addition, the authors also reported a lower prevalence of the herpes simplex virus in those receiving the cash transfer compared to those who did not (0.7% vs 3.0%) [46]. In contrast, a South African study (*n* = 2448, 13–20 years) found no impact of CT on HIV incidence [45]. In Tanzania (*n* = 2720, 15–23 years), UCT paired with combination HIV prevention had no impact on HIV or HSV-2 incidence when compared to combination HIV prevention alone. However, they did find a location-specific (rural) lower incidence of HSV-2 infection in the UCT paired with combination HIV prevention [50]. In the Kenyan study mentioned above, in the older subsample of girls aged 13–14 years at baseline, there were significant reductions in the HSV-2 prevalence (−0.075, 95%CI −0.14;-0.01) and HSV-2 incidence (−0.056, 95%CI −0.11;0.00) in the VE arm, but not in the VEH or VEHW groups, when compared to the V-only group [40].

#### Sexual activity or behaviour and pregnancy

Over half of the studies included in this scoping review (*n* = 10) reported sexual activity/behaviour, or pregnancy, as a primary outcome. Beauclair et al. (*n* = 2907, 13–22 years) found that CCT encouraged young Malawian women to form age-similar relationships, which resulted in increased condom use and reduced sex frequency [47]. This was similar to a study in South Africa (*n* = 3401, 10–18 years) where the authors found a significant reduction in sexual activity (OR 0.49, 95%CI 0.26–0.93) in adolescent girls receiving a CT compared to those who did not receive a CT [42]. This similar effect on a reduction in sexual activity was observed in Zimbabwean (n-2909, 15–59 years) males and females, where those receiving a CT reduced sexual activity by 11.7% points (95%CI −26.0 to 2.61) and 5.68% points (95%CI −15.70 to 4.34), respectively [53]. A study in Liberia (*n* = 1176, 13–14 years) reported significant positive effects on reproductive and sexual health in adolescents receiving both life skills as well as CT compared to those receiving no intervention [54]. Handa et al. [38], reported that Kenyan adolescents (*n* = 1881, 15–25 years) receiving a UCT had significantly lower odds of having sex compared to their counterparts who did not receive a UCT (aOR 0.69, 95%CI 0.53–0.86) [38]. However, in contrast to the study by Beauclair et al. [47], Handa et al. (*n* = 1547, 12–24 years) found no difference in condom use between the groups. In another Kenyan study, UCT reduced the likelihood of pregnancy by 5.0% (*p* < 0.001) in adolescents and young women [39]. Kangwana et al. [40], (*n* = 2075, 11–14 years) showed that ever having given birth was 2.3% lower (−0.023, 95%CI −0.05;0.00) in girls in the combined VE and VEHW groups compared to the V-only group [40]. Additionally, in a subgroup of participants not enrolled in school at baseline, the VE and VEHW groups show a positive impact on ever married (VE: −0.243, 95%CI −0.39;-0.10, VEHW: −0.168, 95%CI −0.33;0.00) and ever been pregnant (VE: −0.182, 95%CI −0.33;-0.03, VEHW: −0.154, 95%CI −0.31;0.00) [41]. In Zambia (*n* = 4922, grade 7–18 years), sexual activity in the previous four weeks was shown to be lower (RR 0.70, 95%CI 0.54;0.91) in the economic support (ES) (UCT adolescent + UCT parent + conditional school fees (CCT)) arm compared to the control arm, and in the combined ES and community dialogue compared to the ES arm (RR 0.84, 95%CI 0.66;1.08) [51]. Unprotected sexual activity was lower in the ES arm compared to the control (aRR 0.80, 95%CI 0.56; 1.16) and in the combined arm compared to the two other arms (aRR 0.65, 95%CI 0.46;0.92 and aRR 0.53, 95%CI 0.37; 0.75 for combined vs economic and control, respectively) [51]. A higher proportion of girls reported current contraceptive use in the combined group compared to the ES group (RR 1.26, 95%CI 1.06; 1.50) and the control group (RR 1.14, 95%CI 0.95; 1.37) [51]. The proportion of girls reporting recent contraceptive use was similar across the three groups. Modern contraceptive knowledge was similar in the control and ES arms. Compared to ES alone, a higher proportion of the combined group had good knowledge (RR 1.18, 95%CI 1.01; 1.38) [51]. In Tanzania (*n* = 2720, 15–23 years) UCT paired with combination HIV prevention reduced the proportion of transactional sex (aOR 0.84, 95%CI 0.73; 0.96), but no significant difference was seen in compensated and intergenerational sex, sex work, and more than one sexual partner in the last 12 months [50]. Urbanisation however did modify the effect on sex work showing a significant increase in urban areas in the CT groups [50].

#### Partner violence

A study conducted in South Africa (*n* = 2448, 13–20 years) found a positive effect of CCT on a reduction in physical partner violence (RR 0.66, 95% CI 0.59–0.74) [43].

#### Economic and food security

In the study by Kilburn et al. [44], in rural South Africa (*n* = 2448, 13–20 years), the CCT was split, with two-thirds going to the caregiver and one-third to the adolescent girl or young woman. They found that adolescent girls and young women receiving a CCT presented with an overall improved economic well-being as measured by having increased savings, having more spending money during the month, not being in debt, and increased food security [44]. The authors found that the CCT significantly increased both total and food per capita household consumption by 4–5%, leading to a significant increase in the index for the economic well-being of 0.15 points (*p* < 0.001) and led to a larger impact on the economic index (0.16 points, *p* < 0.001) [44]. In Tanzania (*n* = 2720, 15–23 years), an increase in savings was seen in those in the CHP + UCT group compared to the control group (aOR 1.87, 95%CI 1.69; 2.08) [50].

#### Tuberculosis treatment

In Peru (*n* = 282, 5–19 years) Wingfield et al. [55] found that those who received a CCT (standard of care, with a cash transfer on condition of household visits) were two times more likely to start tuberculosis treatment compared to those only receiving standard of care (aOR: 2.2, 95%CI 1.1; 4.1). Overall, 43% of those in the CCT program initiated treatment compared to 25% in the control arm. Furthermore, the authors reported that 64% of those within the CCT had successful treatment compared to 53% who only received standard of care [55].

#### Nutritional status

Only one study, conducted in Kenya (*n* = 1481, 0–18 years), looked at the impact of UCT on nutritional status [37]. The authors found that those receiving a UCT were less likely to have stunting for their age (aOR 0.65, 95%CI 0.47–0.89) [37].

#### Healthcare utilisation

A study conducted in rural Zambia (*n* = 4299, grade 7 through to age 18 years) found that economic support alone (RR 1.0, 95%CI 0.9–1.2) or in combination with community dialogue (RR 1.1, 95%CI 0.9; 1.3) did not impact healthcare utilisation or catastrophic health experiences but did reduce the degree of inequality in outpatient healthcare utilisation and catastrophic health experiences across wealth groups [52]. Conversely, in Tanzania (*n* = 2720, 15–23 years), CHP + UCT increased utilisation of community-based biomedical services (aOR 2.10, 95%CI 1.95; 2.26) when compared to adolescents in the control group [50].

## Discussion and conclusions

This scoping review synthesised evidence from 19 randomised controlled trials published between 2012 and 2025 that incorporated cash transfers (CTs) within adolescent-focused interventions in LMICs. Three overarching patterns emerge. First, the evidence base is geographically concentrated: almost all trials were conducted in sub-Saharan Africa (SSA), with only one study from South America. Second, most studies reported beneficial effects on at least one measured outcome, particularly in domains closely linked to the social determinants of adolescent health (schooling, economic security, sexual and reproductive health, and psychosocial wellbeing). Third, despite the framing of CTs as potentially nutrition-sensitive interventions, nutritional status was rarely measured; only one study evaluated anthropometry as an outcome (height-for-age).

Across studies, CTs (conditional and unconditional) were consistently associated with improvements in outcomes plausibly mediated through reduced material deprivation and altered risk environments. Effects on school attendance/enrolment were among the most consistently positive findings, aligning with the logic of CTs as mechanisms to offset direct and opportunity costs of schooling (fees, transport, uniforms, food) and to increase household liquidity. Schooling effects are also likely to be upstream drivers of downstream health benefits, including reduced sexual risk behaviours, delayed pregnancy, and improved psychosocial outcomes, particularly when CTs are explicitly conditional on attendance or embedded in multi-component platforms.

Intervention design varied considerably across studies and appeared to influence outcomes. Most programmes were implemented over one to three years, with longer and multi-component interventions generally showing stronger effects on behavioural and reproductive health outcomes. Transfer amounts were typically modest and paid monthly or bi-monthly, but larger transfers did not consistently produce improved outcomes. Some interventions provided transfers directly to caregivers or households, while others explicitly targeted adolescents through direct payments or conditions linked to adolescent behaviours such as school attendance. Adolescent-targeted interventions appeared more likely to improve adolescent-specific outcomes, including sexual behaviour, contraceptive use, and school retention, whereas household-targeted programmes more commonly improved broader educational and socioeconomic outcomes. Additionally, interventions combining cash transfers with educational, empowerment, or community-based components often demonstrated broader benefits than cash transfers alone, suggesting that programme design and complementary support may be important determinants of effectiveness.

Evidence for mental health benefits (reduced depressive symptoms; improved psychosocial wellbeing) is notable given the limited availability of adolescent mental health services in many LMIC settings. These effects are biologically and socially plausible: CTs can reduce chronic stressors related to food insecurity, debt, and household instability, and may increase adolescents’ perceived agency and social participation. However, mixed sex and age-specific patterns (e.g. differential effects among older females in one setting) indicate that CTs may interact with gendered risks and expectations, including caregiving burdens, intimate partner dynamics, and social surveillance linked to conditionality.

Over half of the included studies assessed sexual behaviour, pregnancy, or sexually transmitted infections. Many trials reported reductions in sexual activity, transactional sex, or early pregnancy, and some observed reductions in HSV-2/HIV prevalence or incidence in specific subgroups. These findings support the proposition that CTs can reduce reliance on transactional or age-disparate partnerships by easing economic constraints and shifting relationship power dynamics. Importantly, several trials suggest that effects are not uniform: impacts differed by sex, age, baseline school enrolment, and urban – rural context. The Tanzanian findings indicating potential effect modification by urbanisation, alongside subgroup-specific impacts in Kenyan trials, reinforce that local sexual economies, labour markets, and normative expectations can shape both pathways and unintended consequences. The Tanzanian findings indicating potential effect modification by urbanisation [50] alongside subgroup-specific impacts in Kenyan trials [40] reinforce that local sexual economies, labour markets, and normative expectations can shape both pathways and unintended consequences. This highlights the need to interpret CT effects as contingent on context, implementation design, and the broader social environment, rather than as universally transferable ‘silver bullets’.

Only one study reported intimate partner violence outcomes, showing reductions in physical partner violence [43]. This aligns with broader conceptual models whereby economic strengthening and reduced financial dependency can lower vulnerability to violence, especially when CTs reach adolescent girls directly or shift household bargaining power. Yet the paucity of trials measuring violence and safety outcomes is a critical gap given the high prevalence and strong health consequences of adolescent exposure to violence. Future trials should incorporate harmonised violence measures and explicitly test mechanisms (economic autonomy, school attendance, relationship dynamics, alcohol use, and community norms).

Although the rationale for CTs often includes improving health through poverty alleviation (and by extension, diet quality and food security), adolescent nutritional status and dietary outcomes were largely absent. Only one trial assessed anthropometry (stunting) across a broad age range, limiting inference for adolescents specifically. This gap is striking for two reasons. First, adolescence is a period of increased nutrient requirements and vulnerability to both undernutrition and emerging overweight/obesity in settings undergoing nutrition transition [56]. Second, CTs could plausibly influence diet quality (diversity, ultra-processed food consumption, meal frequency), but effects may be directionally ambiguous if healthier foods are less accessible or if food environments promote inexpensive energy-dense products [20]. The review, therefore, suggests that the current RCT literature has evaluated CTs primarily as tools for education and HIV/sexual risk reduction, with limited attention to nutrition as a primary endpoint.

The evidence base included conditional CTs, unconditional CTs, and mixed designs, but the review suggests that ‘CT’ often functions as an umbrella term masking meaningful heterogeneity: recipient (caregiver vs adolescent), split transfers, transfer size and frequency, duration, delivery mechanisms, and complementary components (life skills, HIV prevention, community dialogue, violence prevention). Where reported, design features appear to matter. For example, splitting transfers between caregivers and adolescents plausibly supports both household consumption and adolescent agency, and multi-component programmes often show broader effects than CT alone. Conversely, conditionality may enhance schooling outcomes but could generate stress, exclusion, or gendered burdens in contexts where compliance is difficult. These design considerations should be foregrounded in interpretation: ‘CT effectiveness’ is not one intervention but a family of interventions with different causal pathways.

Taken together, the reviewed trials indicate that CTs can improve several upstream determinants of adolescent health, particularly schooling, economic well-being, and selected sexual and reproductive health outcomes, suggesting CTs are credible components of adolescent health strategies in LMICs. However, translation into policy should be cautious and purposeful:

1. Position CTs as part of a package, not a standalone solution, especially where outcomes depend on complementary services (sexual and reproductive health access, violence prevention, mental health support, or nutrition counselling).
2. Match CT design to outcome goals. If education retention is primary, conditionality and school-linked delivery may be appropriate; if mental health or protection is primary, unconditional or less punitive approaches may be preferable.
3. Plan for equity and context, as there may be differential effects by gender, baseline enrolment, and urbanisation.

Although findings were presented according to outcome domains for clarity, many observed effects likely operate through interconnected pathways related to food security, care environments, and broader household and social determinants.

This review highlights a clear agenda for future studies. To strengthen comparability and cumulative learning, researchers should standardise and report core intervention characteristics (transfer value, frequency and duration, recipient, conditionality and enforcement, and implementation fidelity) and adopt harmonised outcome measures to enable robust meta-analysis. Longer follow-up is also needed to assess sustainability after transfers end and to quantify spillover effects within households and communities. Given the current geographic concentration of trials in SSA, expanding evaluations to other LMIC regions, particularly South and Southeast Asia, the Middle East, and Latin America, would improve external validity in contexts where adolescent nutrition transitions and CT platforms are well established. Finally, alongside benefits, studies should systematically assess potential unintended consequences, including stigma, stress induced by conditionality, intra-household conflict, and context-specific increases in risk behaviours, to inform safer and more equity-oriented programme design.

A key strength is the focus on RCTs in adolescents and the mapping of outcomes across multiple health domains. Nonetheless, scoping reviews do not typically assess risk of bias or pool effect sizes, and the heterogeneity of interventions and outcomes limits causal generalisation beyond the included contexts. In addition, some studies enrolled broad age ranges, including adolescents, potentially diluting adolescent-specific inference. The limited availability of age-disaggregated data constrained our ability to isolate impacts among adolescents alone and underscores the need for future studies to report outcomes separately for this population. Finally, the high proportion of ‘positive effects’ and interpretation of programme effectiveness should be considered cautiously, given the heterogeneity of outcomes assessed across studies. Included trials evaluated diverse domains of adolescent wellbeing, with varying measures, effect sizes, and levels of methodological rigour. Consequently, statistically significant findings in individual studies should not be interpreted as indicating uniformly strong or clinically meaningful impacts across all domains. Future research would benefit from greater consistency in outcome measurement and more attention to the practical and policy relevance of observed effects.

In conclusion, the RCT evidence base indicates that CTs can contribute to improved adolescent wellbeing in LMICs, particularly through pathways related to schooling, economic security, and sexual and reproductive health risk. However, direct evidence on adolescent nutrition outcomes is sparse. Future research should move beyond schooling and HIV endpoints to explicitly test nutrition-sensitive CT designs within real-world food environments, using harmonised measures and longer-term follow-up to guide scalable, equity-oriented policy.

## Supplementary Material

PRISMA ScR Checklist.pdf
